# Subjective Outcome Evaluation of the Project P.A.T.H.S.: Qualitative Findings Based on the Experiences of Program Implementers

**DOI:** 10.1100/tsw.2007.161

**Published:** 2007-06-22

**Authors:** Daniel T. L. Shek, Rachael C. F. Sun

**Affiliations:** ^1^ Centre for Quality of Life, Hong Kong Institute of Asia-Pacific Studies, The Chinese University of Hong Kong, China; ^2^ Kiang Wu Nursing College of Macau, China; ^3^ Social Welfare Practice and Research Centre, Department of Social Work, The Chinese University of Hong Kong, Shatin, Hong Kong, China

**Keywords:** subjective outcome evaluation, open-ended questions, qualitative data, program implementers, positive youth development

## Abstract

A total of 52 schools participated in the experimental implementation phase of the project P.A.T.H.S. (Positive Adolescent Training through Holistic Social Programmes). After completion of the Tier 1 Program (Secondary 1 level), 344 teachers and social workers responded to the Subjective Outcome Evaluation Form (Form B), assessing their views of the program and their own performance. Qualitative data analyses based on the schools' evaluation reports showed that the program implementers had enhanced knowledge and skills, learned to establish instructor-student relationships and cooperate with colleagues, and fostered self-development. The workers also appreciated the program philosophy and values, program design and resources, process of implementation, interaction between instructors and students, and program effectiveness. The findings also revealed that the workers encountered difficulties in the program implementation and they also made suggestions on how the program design, program arrangement, manpower deployment, and support for the program implementation could be improved.

## INTRODUCTION

The client satisfaction approach or subjective outcome evaluation is a commonly adopted strategy in program evaluation. Evaluators usually gauge the degree of dissatisfaction and perceptions of program benefits from the perspective of the participants[1,2]. Although it is perfectly legitimate to assess perceived satisfaction from the perspective of the program participants, it is equally important to take the perspective of the program implementers into account. Based on the perspective of utilization-focused evaluation[3], Lilja et al.[4] argued that "to achieve more reliable and valid evaluations, a number of data sources and perspectives should be combined. This means it is necessary to get data which describe the process and the results from both the client's and therapist's perspectives" (p. 1225).

Unfortunately, there are few empirical studies assessing the subjective viewpoints from the program implementers' perspective[5], particularly in the field of positive youth development programs[6]. In their examination of the subjective perceptions of the program implementers on a positive youth development program, Shek et al.[7] argued that: (a) workers' views should be heard and respected alongside those of the program participants, (b) workers have professional skills and experiences to give a more accurate assessment of program effectiveness, (c) there is a need to encourage reflective practice in helping professionals, (d) there is a need to build up a systematic profile of workers' experiences in order to reduce rumors about the program implementation, and (e) it is important to use different data sources (program participants and implementers) to triangulate the results and to rule out biases and errors based on any one source of data. Hence, it is expected that including workers' perceptions and experiences, as well as those of clients, in program evaluation would obtain a complete picture on program usefulness[8].

An examination of the existing literature on client satisfaction studies showed that most of these subjective outcome evaluation studies assessed participants' satisfaction and their perception of program helpfulness through closed-ended questionnaires[1,9] and/or open-ended questions[2,10]. In fact, both formats of data collection have their advantages and disadvantages. For example, although closed-ended rating scales are easy to administer, they have been criticized for their inability to explore the inner world of the respondents, due to the predetermined questions and limited response format[11,12,13]. On the other hand, open-ended questions have been queried of their cost-effectiveness, but Barber et al.[14] argued that "qualitative approaches are more resource-intensive but access in-depth information not captured by reductionist, structured questionnaires" (p.18). Therefore, the importance of using open-ended questions to understand the experiences and perceptions of the program participants has been advocated[15,16,17].

Based on the above arguments, apart from using rating scales to assess program implementers' satisfaction of a program[5], it is equally important to use qualitative methods such as open-ended questions to collect personal views of program implementers. For example, Godley and colleagues[18] used open-ended questions to interview therapists and case managers on their experiences of using manuals as a treatment guide for adolescent marijuana users. Fortune[19] also used both closed- and open-ended questions to interview social workers on the termination reactions they and their clients experienced. In the area of positive youth development, it is noteworthy that there is a paucity of studies using survey questionnaires with open- as well as closed-ended questions in gauging program implementers' subjective views.

In the Project P.A.T.H.S. (Positive Adolescent Training through Holistic Social Programmes), the Tier 1 Program is a universal positive youth development program provided for Secondary 1 to 3 students in Hong Kong. There were 52 schools joining the experimental implementation phase (Secondary 1 level) in the school year 2005/06; 29 of them adopted the full program (i.e., 20-h program involving 40 units) and 23 adopted the core program (i.e., 10-h program involving 20 units).

Several studies have already documented the positive program effects based on the students' objective and subjective outcomes collected from survey questionnaires[20,21,22], as well as the students' subjective experiences revealed in the interviews[23]. In order to gauge the views of the program implementers (i.e., teachers and social workers), telephone interviews, focus groups, and questionnaire surveys were also conducted. In the study that used telephone interviews to collect the program implementers' views of the program[24], although positive program effects were reported, the sample included just 28 teachers and social workers implementing the project in 27 schools, although the respondents were recruited from more than half of the participating schools. Also, it was a process evaluation rather than an outcome evaluation because the interviews were conducted in the midst of the program implementation.

After completion of the Tier 1 Program, all program implementers of the 52 participating schools were required to respond to the Subjective Outcome Evaluation Form (Form B) where both quantitative and qualitative data were collected. Based on the evaluation reports on the consolidated subjective outcome evaluation profile of the participating schools, Shek et al.[7] aggregated the quantitative subjective outcome evaluation data and reconstructed an overall profile of the perceptions and experiences of the program implementers. The quantitative evaluation findings basically revealed that the program implementers had positive perceptions of the program and their own performance, and regarded the program as helpful to the students. On top of these quantitative findings, it would be enlightening if qualitative findings based on the open-ended questions could be examined.

In the present paper, qualitative analyses of the school evaluation reports submitted based on a general qualitative orientation[25] were conducted to answer the following research questions:

- What have the instructors learned in the implementation process?
- What were the things the instructors appreciated most in this program?
- What difficulties did the instructors encounter when implementing this program?
- What aspects of the program should be improved?

The primary purpose of asking these questions was to supplement the quantitative findings by qualitative findings from the program implementers' perspective, so as to evaluate the program usefulness as well as to give insight for future improvement. The secondary purpose was to encourage the program implementers to be reflective practitioners who would reflect on the implementation process (e.g., listing out what they had learned and describe the difficulties encountered) because the Project P.A.T.H.S. places a strong emphasis on the positive development of the program implementers as well as that of the program participants.

## METHODS

### Participants and Procedures

There were 52 schools joining the Project P.A.T.H.S. 2005/06 experimental implementation phase. The mean number of students per school was 166.90 (range: 37–240 students), with an average of 4.58 classes per school (range: 2–7 classes). The mean numbers of social workers and teachers implementing the program per school were 2.63 (range: 0–8) and 5.13 (range: 0–17), respectively.

After the Tier 1 Program (Secondary 1 level) was completed, the program implementers were invited to respond to a subjective outcome evaluation questionnaire. A total of 344 teachers and social workers responded to the Subjective Outcome Evaluation Form (Form B) developed by the research team. An evaluation manual with standardized instructions for collecting the subjective outcome evaluation data was developed[26], and adequate training was provided to the workers during the 20-h training workshops on how to collect and analyze the data collected by Form B.

### Instruments

The Subjective Outcome Evaluation Form (Form B) was designed by Daniel Shek and Andrew Siu[26]. Broadly speaking, there are several parts in this evaluation form as follows:

- Program implementers' perceptions of the program, such as program objectives, design, classroom atmosphere, interaction among the students, and the respondent's participation during class (10 items)
- Program implementers' perceptions of their own practice, including their understanding of the course, teaching skills, professional attitude, involvement, and interaction with the students (10 items)

- Program implementers' perceptions of the effectiveness of the program, such as promotion of different psychosocial competencies, resilience, and overall personal development (16 items)
- The extent to which the program implementers would recommend the program to other students with similar needs (1 item)
- The extent to which the program implementers would teach similar programs in future (1 item)
- Overall satisfaction with the program (1 item)
- Things that the program implementers learned from the program (open-ended question)
- Things that the program implementers appreciated most (open-ended question)
- Difficulties encountered (open-ended question)
- Aspects of the program that require improvement (open-ended question)

### Data Analyses

As the Project P.A.T.H.S. was financially supported by the Hong Kong Jockey Club Charities Trust, each participating school was required to submit an evaluation report to the funding body, together with the consolidated subjective outcome evaluation profile of the school. After receiving the schools' evaluation reports, the research team aggregated the data to "reconstruct" the overall profile based on the subjective outcome evaluation data. The present study focused on the four open-ended questions only and conducted secondary data analyses. The data were analyzed using general qualitative analyses techniques[27] by a registered social worker and a colleague with a Master's degree in Social Work. There were three steps in the process. First, relevant raw codes were developed for words, phrases, and/or sentences that formed meaningful units at the raw responses level. Second, the codes were further combined to reflect higher-order attributes at the category of codes level. Third, the categories of codes were further analyzed to reveal the broader themes at the thematic level. For example, the response that instructors had learned "using teaching materials" at the raw response level could be subsumed under the category of "teaching methods and skills", which could be further subsumed under the broad theme of "enhancement of skills" (see Table 1). Following the principles of qualitative analyses[25], the raw data and categorized data were kept in by a systematic filing system in order to ensure that the findings are auditable.

In the present qualitative analyses, as the researchers designed the program in the P.A.T.H.S. project, they were conscious of their own biases and expectation on the program to be effective. In addition, in order to minimize the possible biases involved, both intra- and inter-rater reliability on the coding was calculated. For intrarater reliability, two colleagues who coded the responses were invited to individually code 20 randomly selected responses for each question. For inter-rater reliability, a research assistant with a Master's degree coded 20 randomly selected responses for each question without knowing the original codes given at the end of the scoring process with reference to the codes finalized by the first author. Also, the second author and a doctoral student performed inter-rater reliability on the first two and last two questions, respectively.

## RESULTS

As shown in Table 1, a total of 174 meaningful units in six categories (i.e., enrichment of knowledge, enhancement of skills, instructor-student relationship, cooperation between colleagues, development of instructors, and others) were formed to indicate the things that the program implementers learned in the implementation process. Most of the respondents reported that they had learned to build up "instructor-student relationship" (n = 48), such as "to understand the students more". Some of them had enriched the "knowledge about adolescents" (n = 26) and enhanced "teaching methods and skills" (n = 21), such as "diverse teaching methods". The percentages of intrarater agreement were 90 and 100%, and inter-rater agreement percentages were 95 and 95%.

**TABLE 1**
**Summary of the Program Implementers' Comments on the Question: "What have the instructors learned in the implementation process?"**

| Category | Subcategory | Codes | N | N in Subcategory |
|---|---|---|---|---|
| Enrichment of knowledge | Knowledge about adolescents | Had a deeper understanding on the issues related to youth development | 10 | 26 |
| | | Understood adolescents' needs | 16 | |
| | Knowledge about China | Knew more about China | 2 | 2 |
| Enhancement of skills | Curriculum design | Developed the skills of transforming theory to a practical curriculum | 2 | 12 |
| | | Enhanced the skills of designing a curriculum | 10 | |
| | Teaching methods and skills | Diverse teaching methods | 7 | 21 |
| | | Enhanced teaching skills | 12 | |
| | | Used teaching materials | 2 | |
| | Facilitating skills | Enhanced skills of leading small groups | 3 | 8 |
| | | Enhanced skills of facilitating discussion | 5 | |
| | Classroom management | Enhanced classroom management | 9 | 9 |
| | Communication and counseling skills | Enhanced communication and counseling skills | 3 | 3 |
| Instructor-student relationship | | Established a good instructor-student relationship | 10 | 48 |
| | | Enhanced interaction | 9 | |
| | | Understood students more | 26 | |
| | | Increased interaction between instructors and students | 3 | |
| Cooperation between colleagues | | Whole school collaboration | 1 | 4 |
| | | Increased interaction with other colleagues (teachers/social workers) | 3 | |
| The development of instructor | Self development | Had more personal reflections | 12 | 17 |
| | | More initiative | 3 | |
| | | Had more confidence and found inspiration to teach | 2 | |
| | Positive views on students | Perceived students in a more positive way | 12 | 12 |
| Others | | Lightened workload | 1 | 12 |
| | | Others | 8 | |
| | | No comment/nothing had been learned | 3 | |
| | | **Total (N)** | **174** | |

*Note:* 20 raw descriptors were randomly selected and coded for examining intra- and inter-rater reliability. The colleagues had to code the randomly selected descriptors into five categories (i.e., enrichment of knowledge, enhancement of skills, instructor-student relationship, cooperation between colleagues, the development of instructors) without knowing the original codes given.

The things that program implementers appreciated most in the program are shown in Table 2. A total of 206 meaningful units in six categories (i.e., program philosophy and values, program design and resources, process of implementation, interaction between instructors and students, program effectiveness, and others) were categorized. The most appreciated areas were program design and resources in term of "program content" (n = 49, e.g., "fit to daily life and reality"), "program materials" (n = 29, e.g., "teaching manuals were user friendly"), and "program arrangement" (n = 29, e.g., systematic program arrangement). The next appreciated areas were the "program philosophy and values" (n = 42, e.g., program rationales) and "program effectiveness" (n = 35, e.g., increased students' participation). The percentages of intrarater agreement were 95 and 100%, and inter-rater agreement percentages were 100 and 95%.

**TABLE 2**
**Summary of the Program Implementers' Comments on the Question: "What were the things the instructors appreciated most in this program?"**

| Category | Subcategory | Codes | N | N in Subcategory |
|---|---|---|---|---|
| Program philosophy and values | | Program rationales | 16 | 42 |
| | | Theoretical bases of the program | 12 | |
| | | Clear program objectives | 9 | |
| | | The efforts and ideas of curriculum designers | 3 | |
| | | The emphasis on students' positive development | 2 | |
| Program design and resources | Program content | Program content and scope | 22 | 49 |
| | | Program design | 16 | |
| | | Fit to daily life and reality | 11 | |
| | Program materials | Teaching manuals were user friendly | 4 | 29 |
| | | Teaching materials | 25 | |
| | Program arrangement | Systematic program arrangement | 22 | 29 |
| | | Sufficient resources | 7 | |
| Process of implementation | Involvement | Students' active participation | 5 | 11 |
| | | Instructors were involved | 6 | |
| | Teaching process | Enriched teaching skills and experiences | 1 | 4 |
| | | Reduced preparation time | 2 | |
| | | Had preprogram training for instructors | 1 | |
| Interaction between instructors and students | | Increased interaction between instructors and students | 5 | 5 |
| Program effectiveness | | Enhanced adolescents' development | 8 | 35 |
| | | Increased students' participation | 19 | |
| | | Had effectiveness | 8 | |
| Others | | No comment | 1 | 2 |
| | | Nothing | 1 | |
| | | **Total (N)** | **206** | |

*Note*: 20 raw descriptors were randomly selected and coded for examining intra- and inter-rater reliability. The colleagues had to code the randomly selected descriptors into 5 categories (i.e., program philosophy and values, program design and resources, process of implementation, interaction between instructors and students, program effectiveness) without knowing the original codes given.

As shown in Table 3, 254 difficulties encountered in program implementation were abstracted from the findings, which were classified into five categories (i.e., program design and resources, course arrangement, process of implementation, students' characteristics, and others). Most of the respondents raised difficulties encountered in the "program content" of the program design and resources (n = 65, e.g., program content was too much), and in dealing with the "time issues" in course arrangement (n = 43, e.g., insufficient time for each session). The percentages of intrarater agreement were 95 and 100%, and inter-rater agreement percentages were 90 and 90%.

As shown in Table 4, 233 codes for improvement were recorded, which were classified into five categories (i.e., program content, program arrangement, arrangement of manpower, support for the program, and others). Most of the respondents suggested that the "program content or format needs to be strengthened" (n = 90, e.g., make the teaching materials or activities more interesting), and at the same time suggested that the "program content or format needs be trimmed" (n = 47, e.g., simplify program content), and pointed out that the "time issues" (n = 31) in program arrangement needed to be dealt with (e.g., regulate the number of session and time according to program content). The percentages of intrarater agreement were 90 and 100%, and inter-rater agreement percentages were 95 and 90%.

## DISCUSSION

There are several observations based on the present findings. First, workers felt that they had learned many things in the program (Table 1) and they appreciated different aspects of the program (Table 2). Second, difficulties encountered by the workers (Table 3) and suggestions for improvement (Table 4) were raised by the respondents. Third, there were different views on the program design and resources. While there were 149 responses endorsing the program philosophy and values as well as program design and resources (see Table 2), there were 79 difficulties (see Table 3) and 137 suggestions for improvement (see Table 4). In other words, different workers had different perceptions of these areas. Fourth, as shown in Table 3, most of the difficulties raised were related to program implementation (175 responses) rather than the program design (79 responses). Fifth, in conjunction with the quantitative subjective outcome evaluation data[7], the picture that can be derived is that most of the participants and workers were positive about the program and they perceived the benefits of the program to the participants, although there were also views indicating difficulties encountered and suggestions for future improvement. As the data were collected at the first year of the experimental implementation phase, it is reasonable to expect the program implementers encountered difficulties in program implementation.

Regarding the difficulties faced by the program implementers, several salient observations could be highlighted. First, time management was the most commonly mentioned difficulty in program implementation and program implementers voiced that there was insufficient time for completing the teaching units. Although the teaching units had been field tested and the program implementers should have been able to complete the related activities within the suggested time, this concern was taken into account in the manuals developed for the full implementation phase. By categorizing the developed activities of a teaching unit into primary and secondary activities, the program implementers would have felt less psychological burden. Practically speaking, it is noteworthy that workers should arrange sufficient time to teach each unit, and avoid dealing too frequently with class administration matters in the same lesson.

Second, poor time arrangement was the next difficulty that may be due to the tight school timetable. The school administrator could only arrange after-school lessons or Saturday mornings to run the Tier 1 Program, thus resulting in low student motivation and staff morale. Fortunately, as reflected by the experiences of some schools, this problem can be minimized if the schools could incorporate the P.A.T.H.S. program into the school's formal curriculum, such as Liberal Studies and Integrated Humanities. Third, some respondents pointed out that they had difficulties in managing class discipline.

**TABLE 3**
**Summary of the Program Implementers' Comments on the Question: "What difficulties did the instructors encounter when implementing this program?"**

| Category | Subcategory | Codes | N | N in Subcategory |
|---|---|---|---|---|
| Program design and resources | Program content | Unclear program objectives | 2 | 65 |
| | | Program content was too much | 23 | |
| | | Topics were overlapping | 6 | |
| | | The curriculum could not cater for students' individual differences | 2 | |
| | | Unable to meet students' needs and match their levels of ability | 12 | |
| | | Program was monotonous | 10 | |
| | | Program arrangement was not good | 10 | |
| | Program materials | Too many worksheets | 3 | 14 |
| | | Teaching materials and program information were insufficient | 6 | |
| | | Unable to utilize "Growth Puzzles" effectively | 5 | |
| Course arrangement | Time issues | Poor time arrangement for the program | 5 | 43 |
| | | Insufficient time for each session | 38 | |
| | Physical setting | Classroom was too small | 3 | 7 |
| | | Too many students | 4 | |
| Process of implementation | Instructor | Insufficient manpower | 5 | 12 |
| | | Colleagues could not cooperate well | 2 | |
| | | Instructors lacked experiences and skills | 5 | |
| | Workload | Increased workload | 15 | 22 |
| | | Insufficient time for preparation | 7 | |
| | Time management | Difficult to manage time | 6 | 6 |
| | Classroom management | Difficult to manage class discipline | 21 | 21 |
| | Teaching process | Difficult to carry out the designed program format | 2 | 17 |
| | | Difficult to carry out discussion | 4 | |
| | | Difficult to facilitate students' reflection | 3 | |
| | | Difficult to maintain students' learning interest | 4 | |
| | | Difficult to carry out the program interactively | 4 | |
| Students' characteristics | | Students were not involved | 13 | 27 |
| | | Students did not have much interest in the program | 7 | |
| | | Differences in students' performance | 7 | |
| Others | | Instructors felt mentally and physically exhausted | 1 | 20 |
| | | The equipment was broken down during the lesson | 1 | |
| | | Difficult to demonstrate teaching effectiveness | 4 | |
| | | Suggestions | 5 | |
| | | Others | 9 | |
| | | **Total (N)** | **254** | |

*Note*: 20 raw descriptors were randomly selected and coded for examining intra- and inter-rater reliability. The colleagues had to code the randomly selected descriptors into four categories (i.e., program design and resources, course arrangement, process of implementation, students’ characteristics) without knowing the original codes given.

**TABLE 4**
**Summary of the Program Implementers' Comments on the Question: "What aspects of the program should be improved?"**

| Category | Subcategory | Codes | N | N in Subcategory |
|---|---|---|---|---|
| Program content | Program content/format needs to be strengthened | Program objectives should be more accurate | 3 | 90 |
| | | Program should be more in depth | 13 | |
| | | Content should be close to daily life | 10 | |
| | | Make the teaching materials or activities more interesting | 25 | |
| | | Improve individual program units | 6 | |
| | | Add or amend supplementary information | 8 | |
| | | Improve supplementary activities | 2 | |
| | | Improve the arrangement of grouping | 2 | |
| | | Improve the design of "Growth Puzzles" | 10 | |
| | | Provide or improve audio-visual teaching materials | 9 | |
| | | Print student manuals | 2 | |
| | Program content/format needs to be trimmed | Simplify program content | 22 | 47 |
| | | Reduce program content or activities | 8 | |
| | | Reduce themes or program units | 5 | |
| | | Decrease the quantity of worksheets or activities which need hand writing | 12 | |
| Program arrangement | Time issues | Regulate the number of sessions and time according to program content | 3 | 31 |
| | | Extend the duration of each session | 26 | |
| | | Earlier preparation before program implementation | 2 | |
| | Physical setting | Change the venue of lessons | 6 | 8 |
| | | Use small class teaching | 2 | |
| | School administration | Improve administrative arrangement | 1 | 26 |
| | | Incorporate the program into formal curriculum | 3 | |
| | | Improve the teaching flow | 3 | |
| | | Increase the flexibility of curriculum and teaching materials | 11 | |
| | | Enhance instructors' autonomy | 8 | |
| Arrangement of manpower | | Increase the number of instructors | 4 | 9 |
| | | Decrease the number of instructors | 1 | |
| | | Assisted by social workers | 3 | |
| | | Taught by teachers | 1 | |
| Support for the program | Knowledge sharing | Web-based sharing platform | 1 | 4 |
| | | Peer sharing among coworkers | 3 | |
| | Additional resources | Strengthen instructors' training | 4 | 5 |
| | | Add resources | 1 | |
| Others | | Other suggestions/opinions | 12 | 13 |
| | | No comment | 1 | |
| | | **Total (N)** | **233** | |

*Note*: 20 raw descriptors were randomly selected and coded for examining intra- and inter-rater reliability. The colleagues had to code the randomly selected descriptors into four categories (i.e., program content, program arrangement, arrangement of manpower, support for the program) without knowing the original codes given.

According to Wolfgang[28], students have some social goals to be attained in the classroom (e.g., a sense of belonging, fun, and power), and they will misbehave if these goals are not satisfied. Therefore, instructors should establish classroom rules at the beginning of the program and enforce such rules by using logical consequences instead of punishment. Also, instructors should promote a sense of ownership and collaborative spirit in class, and the workers should recognize the positive behavior of the students. Fourth, some respondents listed difficulties in the teaching process that were related to a lack of skills in running activities. As didactic learning (i.e., students learn from the teachers via impartation of skills) is the expected form of learning and experiential learning emphasized in Projects P.A.T.H.S. requires a more egalitarian relationship between the teacher and the student that is not common in the Chinese culture, we have offered 20-h training workshops for both teachers and social workers in order to equip them with the necessary skills needed for running the programs. Furthermore, it is hoped that by learning from the experiences in the first year of the experimental implementation phase, the schools and social work agencies could have better arrangement and improvement in administrating the program, and the program implementers could have advanced skills and knowledge and higher levels of resilience in handling and overcoming the difficulties in the coming years.

It is noteworthy that by asking questions on "what difficulties did you encounter in implementing this program?" and "what aspects of the program should be improved?", the research team was actually inviting the workers to express views that might be less emotionally aroused than the wording "are you dissatisfied with the program?", and they encouraged the participants to reveal their inner negative feelings[29]. As such, the statements of problems encountered and suggestions for improvement do not necessarily imply that the participants were not satisfied with the program. In fact, the quantitative findings based on the instructors clearly show that a majority of the instructors were satisfied with the program[7].

The present findings constitute important additions to the literature on the evaluation of positive youth development programs because there is a paucity of research studies examining program implementers' satisfaction and subjective views[5], particularly in positive youth development[7]. In conjunction with the previous findings, the present study clearly revealed that while the program implementers had positive ratings in program, program usefulness to students, and one's performance based on the closed-ended questions, different perceptions and experiences of the workers could be shown in the qualitative findings.

Shek and Siu[30] listed 12 principles in the evaluation of Project P.A.T.H.S. Except Principles 1, 2, and 10, which primarily cover objective outcome evaluation, the present study satisfied the remaining nine principles by conducting a subjective outcome evaluation (Principle 3), inclusion of the workers as one of the stakeholders (Principles 4 and 5), use of qualitative evaluation methods (Principles 6 and 7) on top of quantitative evaluation methods, use of different data sources (program participants and implementers) and data type (quantitative and qualitative data) to triangulate the results and to rule out biases and errors in any one type and source of data (Principles 8 and 9). Finally, existing guidelines on evaluation were adhered to (Principle 11) and collection of subjective outcome evaluation findings from the workers was part of the continuous and ongoing evaluation (Principle 12).

In conclusion, the present qualitative findings based on the experiences of program implementers showed that the respondents had gains from the program, showed appreciation to the program, and revealed difficulties in the process and suggestions for further improvement at the same time. These findings, together with the quantitative findings based on the experiences of program implementers, as well as both the qualitative and quantitative findings based on the experiences of program participants, painted a whole picture of the effectiveness of the Tier 1 Program (Secondary 1 level) of Project P.A.T.H.S.

## ACKNOWLEDGMENTS

The preparation for this paper and the Project P.A.T.H.S. were financially supported by the Hong Kong Jockey Club Charities Trust.
